# Uncertainty Monitoring by Young Children in a Computerized Task

**DOI:** 10.6064/2012/692890

**Published:** 2012-06-14

**Authors:** Michael J. Beran, Scott Decker, Allison Schwartz, J. David Smith

**Affiliations:** ^1^Georgia State University, Language Research Center, Atlanta, GA 30302, USA; ^2^Department of Psychology, University of South Carolina, Columbia, SC 29208, USA; ^3^Department of Counseling and Psychological Services, Georgia State University, Atlanta, GA 30302, USA; ^4^Department of Psychology, University at Buffalo, SUNY, Buffalo, NY 14260-4110, USA

## Abstract

Adult humans show sophisticated metacognitive abilities, including the ability to monitor uncertainty. Unfortunately, most measures of uncertainty monitoring are limited to use with adults due to their general complexity and dependence on explicit verbalization. However, recent research with nonhuman animals has successfully developed measures of uncertainty monitoring that are simple and do not require explicit verbalization. The purpose of this study was to investigate metacognition in young children using uncertainty monitoring tests developed for nonhumans. Children judged whether stimuli were more pink or blue—stimuli nearest the pink-blue midpoint were the most uncertain and the most difficult to classify. Children also had an option to acknowledge difficulty and gain the necessary information for correct classification. As predicted, children most often asked for help on the most difficult stimuli. This result confirms that some metacognitive abilities appear early in cognitive development. The tasks of animal metacognition research clearly have substantial utility for exploring the early developmental roots of human metacognition.

## 1. Introduction

 Humans often have to make decisions in situations in which information is incomplete, or in situations in which they do not know the best course of action. In the former case, they may request more information. In the latter case, they may choose not to choose. That is, they may decline to make a response because they know they do not know the answer. This ability, to know when one knows an answer and when one does not, is called metacognition, and it is sometimes described as “thinking about thinking” [1]. The metacognition shown by adult humans may be intimately connected to important aspects of reflective mind, including cognitive control, self-awareness, and consciousness. It is one of humans' most sophisticated cognitive abilities. Therefore, understanding the earliest developmental roots of metacognition is a fundamental goal of cognitive developmental research.

 At present, the evidence suggests that metacognition appears to emerge quite late in cognitive development [2, 3]. Therefore, its full manifestation could even possibly be a uniquely *adult* human capacity. Children typically do not show many metacognitive abilities until late preschool ages [4–8]. For example, consider a task in which children are asked to predict their performance. Third grade children have been reported as more proficient than first-grade children when predicting which picture-label matches they would be able to recognize [9]. Young children tend to be overconfident relative to older children [3]. They also confuse metacognitive estimates with actual memory contents [10]. But, this does not mean that young children have no way of evaluating information. They seem to be able to tell good information from bad information [11]. Children as young as 2.5 years of age also can indicate when they do or do not know the meaning of a word (e.g., [12]), and children as young as five years of age report higher confidence levels for correct responses than for incorrect ones (e.g., [13]).

 So, we may not yet know the full story regarding young children's metacognitive competence. It may be that younger children struggle to show metacognitive competence because of the nature of the tasks that are used to test them. Reliance on verbal, introspective, and declarative and language-based procedures may explain why younger children fail to show metacognitive performances, and this is not the same as a genuine lack of the underlying capacity (see [8, 14, 15], for extensive discussion of this issue). Some researchers argue that metacognition may be available in a form that allows for implicit access to knowledge states before it is in a form that is well differentiated and verbalizable. Young children may behave in ways that reflect metacognition before they are able to report verbally what it is they are doing. In fact, the use of observational methodologies has shown that young children may provide more nonverbal indicators of metacognition (such as eye gaze shifting, gestures, and pauses) than previously thought (e.g., [16]).

 Accordingly, the best way to assess metacognition in young children could be through tasks that are purely behavioral, nondeclarative, and as language free as possible. Although few such measures currently exist for young children, such tasks have been developed in response to the present sharp interest in cross-species studies of metacognition. Metacognition tasks for nonhuman animals (hereafter, animals) are definitionally purely behavioral, non-declarative, and language free. Therefore, tests developed for use with animals could have a high value for assessing metacognition in young children (see [14]).

 Comparative research indicates that some species may be capable of metacognitive processes, particularly the metacognitive process of uncertainty monitoring. In some cases, animals appear to deal with uncertainty adaptively by seeking information or by refusing to make a choice when the risk of error is high (e.g., [17–21]). The problem in this research areas has been that it is difficult to explain the task rules to animals and impossible to elicit anything like the verbal reports of uncertainty that humans can give. So instead, researchers have designed behavioral tasks that have two components. First, researchers make some trials easy but some trials difficult. Second, researchers give animals a response apart from the task's primary discrimination responses that lets them decline to complete any trials they chose. This *uncertainty response* lets animals manage their uncertainty and declare it behaviorally and observably, if they can, because they should recognize difficult trials as risky and error-causing and decline those trials proactively and adaptively.

 For example, when monkeys are trained to classify stimuli that lie along a psychophysical continuum (e.g., discriminating brightnesses, line lengths, or circle sizes), their performance is poorest for the most difficult stimuli near the discrimination's breakpoint. When monkeys are given a third response that allows them to decline the current trial without receiving reward or punishment for a primary response, they can selectively use this response on exactly those trials for which they are at greatest risk of making an incorrect primary response (e.g., [19, 22–24]). Although there is debate about the appropriate interpretive level for these data with animals (e.g., [25–30]), the data reflect clearly that animals avoid exactly those trials on which they are most likely to make errors. To date, an uncertainty response has been included in tests of psychophysical discrimination in the visual, auditory, and temporal domains [19, 23, 24, 31], in tests of list memory, item memory, and spatial memory [21, 32, 33], in tests of two-choice discrimination learning [34], and in tasks involving judgments of quantity [22] and judgments of sameness and difference [35].

 There is research interest in uncertainty monitoring and metacognition in developmental psychology and education more broadly, and great interest in improving those capacities in children [36]. The tasks designed for use with animals can be adapted easily for use with human populations including very young human children [37, 38], whereas tests used to assess adult human metacognition may not be suitable for young children. Tasks designed for animals may offer new avenues for successfully eliciting uncertainty responding, or they may operate as a scaffolding step toward the more verbal and explicit forms of metacognition that are desired in humans. However, these kinds of tasks have only rarely been adapted for use with young children. In one case, 3.5 year-old children performed a paired-associate memory task and were given the option to skip trials if they wanted when given the recognition memory test [14]. Accuracy for accepted items was significantly higher than for skipped items on a subsequent memory task that included all items, and this suggested that children may have had implicit access to their own knowledge states by this age, even if explicit forms of metacognition could not yet be demonstrated [14]. No test using psychophysical discriminations with an attendant uncertainty response has yet been given to children at this age, and if children also succeeded on that kind of task, this would strengthen the argument that young children have access to at least an implicit form of metacognition.

Thus, we presented young children with a task based on the principles of the uncertainty monitoring paradigm used with nonhuman animals. Although the task is inspired by animal research, and it is true that children do not have to make explicit verbal reports, it still requires some degree of verbal instruction. Therefore, the task is not yet language free, at least as used in the present experiment. We created a simple discrimination task and an engaging story to structure that task for children. This allowed us not only to assess the perceptual difficulties that children faced in the discrimination, but also to assess the extent to which they would use an uncertainly response that allowed them to opt out of the discrimination and instead gain outside information about how to classify the stimulus in question. Our prediction was that children might show data patterns in this task consistent with those shown by animals and adult humans in closely analogous tasks. Specifically, we predicted children would use the uncertainty response more often for difficult items in comparison to less difficult items, thus demonstrating adaptive uncertainty monitoring during psychophysical judgments.

## 2. Methods

### 2.1. Participants

Twenty two children (MN = 54.5 months; SD = 6.8 months) were tested. Children were recruited through a university database of available participants, and all children who came into the laboratory completed this test as part of a larger battery of cognitive tests performed using computerized or manual tests. Three children participated in a pilot version of the task to refine the methodology. Their data were excluded from all analyses because of subsequent changes made to the procedure. The research was conducted with approval from the Georgia State University Institutional Review Board. Informed consent was provided by the parents or legal guardians of the children.

### 2.2. Design and Procedure

The task was programmed in Visual Basic and presented on an IBM-compatible laptop computer so that it could be taken to the testing site for children. Key presses were used to record the responses of the children, with small icons mirroring the large response options on the screen affixed to the relevant keys on the keyboard.

**Figure 1:**
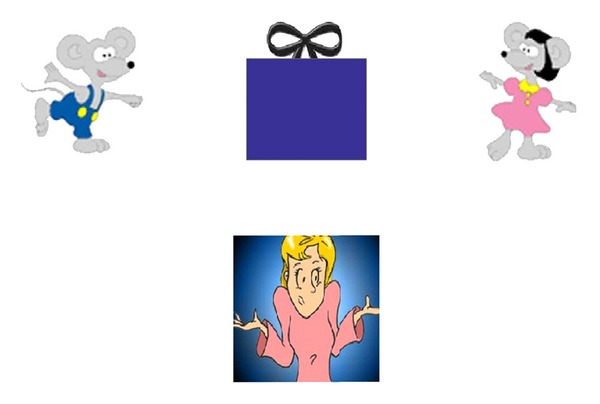
A screen shot of the task. The present is at top center and is shaded in one of 20 shades from bright pink to dark blue. The boy mouse at left is supposed to receive blue presents and the girl mouse is to receive pink presents. The shrugging character (the Helper) at bottom is the uncertainty response stimulus, and when presents are given to her they are then routed to the correct mouse.

Figure 1 shows the uncertainty-monitoring task. On each trial, a stimulus appeared in the screen's top center. It was the digital image of a gift wrapped in a color ranging in 20 steps from the purest blue color to the purest pink color. Half the stimuli were objectively classified as blue and half as pink. The levels closest to the blue-pink breakpoint of the discrimination were expected to be more difficult to classify. A male mouse character—to be the recipient of bluer gifts—was shown at the top left (see Figure 1). A female mouse character—to be the recipient of pinker gifts—was shown at the top right. These two stimuli represented the blue and pink responses in the discrimination. At bottom center was an image of the “helper” shrugging her shoulders. This stimulus represented the uncertainty response.

In the cover story for the task, the help of the child was enlisted to distribute presents to the two mice. The specific instructions given to children were as follows.

*“Today we're going to help our two mice friends who are having a birthday party (point or indicate two mice images). Your job is to make sure each mouse gets the right presents. You need to give the blue presents to the Boy Mouse by pressing this button (point/indicate the blue mouse button) and the pink presents to the Girl Mouse by pressing this button (point/indicate the pink mouse button). It is very important to do your best because when you give the presents to the right mouse a happy face appears and the mouse is happy, but if the wrong mouse gets the present, a sad face will appear because the mouse is sad. If you don't know if the present is pink or blue or who to give it to, don't worry, you can give it to the Helper by pressing this button and she'll find out who the present belongs to. Each time you give 5 presents away, you will earn a sticker to put on your sticker page! If you don't want to push the buttons, tell me who gets the presents and I will do it for you. Are you ready? Let's start handing out the presents!”*

All trials then were initiated by the experimenter with a key press on the keyboard. This was necessary to ensure that the children were ready to attend to the screen and to the trial. Children made responses by key press. The key press to give a present to the male or female mouse caused that present to scroll over to that mouse. The key press to give the present to the helper sent the present scrolling to the helper, at which point it was redirected and scrolled to the correct mouse automatically. Correct responses made by the child produced a smiley face in the center of the screen and a happy chuckling sound. Incorrect responses produced an unhappy face on the screen and a beeping sound. Presents redirected by the helper went to the correct mouse, but there was no auditory or visual feedback beyond that. Children were given as long as they needed to make a response. This was necessary because they showed variable levels of motor skill in pressing the keys, and so a time limit would have precluded many valid choice responses. Additionally, if needed, the children could tell the examiner their response, and then the examiner would push the corresponding key for them.

It is important to note that the stickers were given to ensure that children enjoyed continuing to play the game. Their noncontingent delivery regarding game performance ensured that we were not rewarding use of the uncertainty response, and in fact stickers could have been given immediately after incorrect primary responses if those responses were in the fifth position of a given trial block. It is also important to note that the uncertainty response was available on every trial. Some paradigms that are used with nonhuman animals intersperse trials with or without the uncertainty response that is supposed to reflect metacognition (e.g., [32]), with the goal being to see if the subject is more accurate on trials where they could have chosen to skip the test but did not compared to trials where they were forced to take the test. However, not all paradigms use this method (see [28] for overview), and this is not a feature in the experiments we have given to monkeys, so we used a consistent methodology with the children.

At the beginning of each session, only the two easiest trial levels were presented until the child was correct on 7 of the last 10 trials in classifying these stimuli. These trials were excluded from analyses. Subsequently, all possible levels were presented, with each trial a random selection of one of the 20 levels. Children worked for one session for as long as they were willing to engage the task. The data from two children were excluded due to early discontinuation because they only completed 21 and 18 trials, respectively. All other children completed between 59 and 79 trials (MN = 75.7 trials). In all, the data from 17 children were included for analysis.

## 3. Results

**Figure 2:**
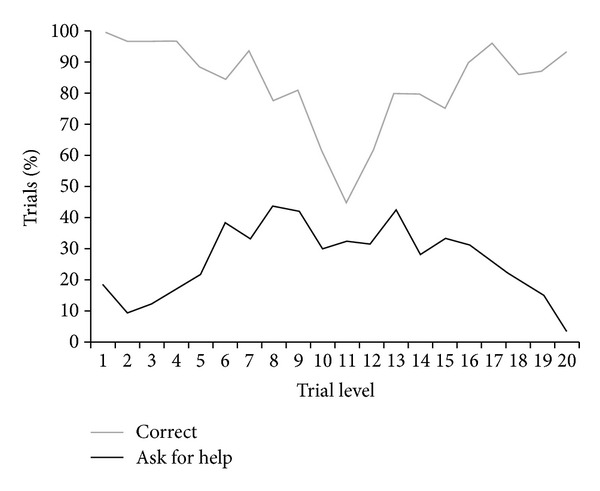
Overall performance (total % trials correct) in assigning the presents to the two mice and the overall percentage of trials at each level for which the children gave the present first to the Helper (the uncertainty response).

Figure 2 shows the performance of the children. The figure shows all trials combined across children to provide the full data set. Analyses of variance were used to assess the relation between stimulus level and the use of the primary responses (boy or girl mouse) and the uncertainty response (the helper). For both curves (percent trials correct and percent trials in which help was requested), the best fit function was quadratic: percent correct *F*(2, 17) = 9.16, *P* < .001; percent help requested *F*(2, 17) = 24.70, *P* < .001. Both curves showed a clear trough or peak near the center of the distribution. In addition, there was a significant negative correlation between the percentage of trials correct and the percentage of trials on which help was requested, *r*(18) = −.49, *P* = .026. This indicated that children were more likely to ask for help on trials for which they were at greater risk of making a classification error when they attempted to distribute the present to either the boy or girl mouse. It should be noted that 15 of the 17 children made sufficiently large numbers of uncertainty responses (10% of trials or more) to make clear that they were willing and able to ask for help. One child made only a single uncertainty response, and one child never used that response.

## 4. Discussion

 In this experiment, we presented young children with a psychophysical discrimination task that included an uncertainty response. The primary discrimination (blue versus pink) was made sufficiently difficult that there were some trial levels on which children would likely make mistakes. Crucially, children were most likely to ask for help—declining to make the classification themselves—on those trial levels. The children's behavioral responses were somewhat similar to adult humans and some nonhuman primate species given similar tests (see [20, 32]). However, performance was not as optimal as usually seen with adults and monkeys. Uncertainty responses were made rather broadly across approximately the middle third of the color range. Children asked for help on a broad range of trials (not just the most difficult ones), and there is certainly room for improvement.

Thus, children evidently experienced uncertainty, monitored that uncertainty, and they responded to that uncertainty adaptively and appropriately using the uncertainty response. These results from a perceptual, psychophysical task complement those from a memory-monitoring task [14]. They support the hypothesis that children younger than five years of age can and sometimes do monitor what they do and do not know. Both lines of research demonstrate that the paradigms originally developed for assessing metacognition in animals have parallel utility for research with young human children. Indeed, those paradigms may be very well suited for tracing the earliest roots of metacognitive abilities during cognitive development, particularly if those paradigms can be validated as reflecting metacognitive states in animals (for this ongoing debate, see [26–30]). It has been a significant problem in developmental research that children have often been given uncertainty monitoring tasks that are novel, difficult, abstract, and that require an explicit verbal response [39]. These tasks may underestimate children's true metacognitive capacities. The present task—familiar, simple, concrete, nonverbal, and perceptual—may provide another profile for revealing uncertainty monitoring in very young children who may be able to enact metacognition procedurally long before they can declare metacognition explicitly. Any child who can respond within a cognitive task might be asked to demonstrate his or her uncertainty-monitoring ability, limited only by the experimenter's ability to frame an intuitive task and a brief/engaging procedure. And so the question remains as to just how young children can be and still show evidence of uncertainty monitoring and metacognition. We hope that more research will be forthcoming in this area.

Procedural metacognition paradigms also have potential for testing metacognition—or fostering metacognition—in populations of autistic, language delayed, or educationally challenged populations. These children regularly fail to sustain and generalize metacognitive activities, and, worse, seem impervious to the training manipulations and remedial programs that seek to foster those capacities, even if those efforts are explicit and extensive [40–43]. To our mind, these interventions have sometimes erred by focusing on metacognition within the complex cognitive tasks of school (e.g., reading comprehension). They also sometimes rely on an explicit, declarative Think Aloud approach (e.g., [44]). This training approach could backfire given the use of complex and verbal tasks, their resource requirements, or a failure of readiness in children to engage in a sophisticated, regulatory dialogue with themselves. A useful complementary approach might be to use paradigms like that used in the present research to give metacognitive strategies like uncertainty monitoring an initial, behavioral, procedural foothold within cognitive functioning. Then, one might bootstrap on the behavioral strategies to broaden their application toward educational activities or gradually help the child bring those strategies into focal consciousness and make them part of explicit, declarative cognition.
